# Older Adults Who Spend More Time Outdoors in Summer and Have Higher Dietary Vitamin D Than Younger Adults Can Present at Least as High Vitamin D Status: A Pilot Study

**DOI:** 10.3390/ijerph18073364

**Published:** 2021-03-24

**Authors:** Oktawia Borecka, Mark D. Farrar, Joanne E. Osman, Lesley E. Rhodes, Ann R. Webb

**Affiliations:** 1 Department of Earth and Environmental Sciences, Faculty of Science and Engineering, University of Manchester, Manchester M13 9PL, UK; ann.webb@manchester.ac.uk; 2 Division of Musculoskeletal and Dermatological Sciences, School of Biological Sciences, Faculty of Medicine Biology and Health, University of Manchester, Manchester M13 9PL, UK; mark.farrar@manchester.ac.uk (M.D.F.); lesley.e.rhodes@manchester.ac.uk (L.E.R.); 3 Photobiology Unit, Dermatology Research Centre, Salford Royal NHS Foundation Trust, Manchester Academic Health Science Centre, Manchester M6 8HD, UK; joanne.osman@manchester.ac.uk

**Keywords:** vitamin D, sunlight exposure, diet, older adults

## Abstract

Vitamin D_3_ can be produced by exposing skin to UVB radiation or sourced through dietary products. It is often stated that vitamin D status declines in older adults, yet little is known about differences in current-day lifestyle and dietary behaviours influencing vitamin D outcomes in younger (18–40 years old) and older adults (65–89 years old). Our objectives were to perform a pilot study to compare sun exposure behaviours, i.e., time spent outdoors, holiday behaviour and use of sunscreen/clothing, and dietary vitamin D intake, in young and older adults in the UK, together with assessment of their vitamin D status. A total of 13 young and 11 older volunteers completed a four-page questionnaire to assess sun exposure and photoprotective behaviour and an eleven-page one-week vitamin D diet diary, alongside their plasma 25(OH)D measurement. It was found that the older group tended to spend more time outdoors during the working week in summer, to take more summer and winter holidays each year, take longer winter holidays and have similar sunscreen use when compared to younger adults. Older adults had a significantly higher daily dietary intake of vitamin D (4.0 μg) than young adults (2.4 μg). Mean winter 25(OH)D concentration was higher in older (56.9 nmol/L) than in young adults (43.2 nmol/L), but there was no statistical difference between the groups. Contrary to common assumptions, in this study, older adults had sun exposure and dietary behaviours conferring a vitamin D status at least as good as that of younger adults.

## 1. Introduction

### 1.1. Vitamin D Production/Intake

Vitamin D is essential for calcium homeostasis, which sustains a healthy musculoskeletal system. Exposure to sunlight allows for the production of vitamin D in human skin. UVB radiation initiates the conversion of the skin precursor 7-dehydrocholesterol (7DHC) to pre-vitamin D and hence metabolites further down the pathway, such as 25-hydroxyvitamin D (25(OH)D) and 1,25-dihydroxyvitamin D (1,25(OH)2D). Serum 25(OH)D concentration is used to assess vitamin D status of a person [1]. It is estimated that 25(OH)D levels below 25 nmol/L (<10 ng/mL) are associated with the diseases of vitamin D deficiency (rickets and osteomalacia) [2] and <50 nmol/L (or <20 ng/mL) with overall poor musculoskeletal health [3,4,5].

In humans, the primary source of vitamin D is considered to be UVB exposure, as dietary sources provide only a fraction of the vitamin D required by the human body [6].

### 1.2. Population Sunlight Exposure Behaviours and Vitamin D Status

Conflicting health messages about the risk/benefit ratio of sunlight exposure can be confusing. In the public eye, the promotion of sun exposure risks is greater than the promotion of benefits, while the understanding of, and adherence to, the ‘little and often’ approach for ensuring adequate vitamin D synthesis is limited [7]. Meanwhile, the production of vitamin D in the body is solely dependent on sunlight exposure, and its lack can have long-term negative health consequences.

Some data indicate that older adults are more prone to having poor vitamin D status [8] due to less time spent outdoors and decreased capacity for cutaneous synthesis [9]. The data from the 1985 study [9], which used surgical specimens, suggest that the ability to synthesise vitamin D decreases with age because of reduced precursor 7DHC. Hence, appropriate adjustments in lifestyle behaviour might be needed in older populations to maintain adequate vitamin D status. However, in the UK, data from older adults are mostly based on research focusing on institutionalised populations [10,11,12] or via older surveys such as National Diet and Nutrition Survey, 1998 [13] and Health Survey for England (HSE) [2], while evidence of decreased vitamin D synthesis in skin is limited [9]. Therefore, as life expectancy increases [14], traditional beliefs regarding behaviour of older adults might no longer be true—a large number of ambulant, non-institutionalised older adults work beyond the age of 65 and take on outdoor activities during retirement [15,16,17]. This shift might have an influence on the vitamin D status of the older population through their adjusted behaviours.

More recent data from UK surveys show that mean plasma 25(OH)D concentrations were not lower in older (65+ years old) compared to younger adults (19–64 years old) and a similar percentage of the groups were deficient in 25(OH)D (<25 nmol/L) [2,18]; on average during the year, 23% of both 19–64 and 65+ year olds [19].

It has been estimated that during the adult life of an indoor worker in the UK, 30% of the annual sunlight exposure comes from sunny holidays, 40% from summer weekends, 20% from summer weekdays and only 10% from exposure between October and March [20]. This significant decrease in the UV exposure during six months of the year explains the observed seasonal cycle in vitamin D status and emphasises the importance of safe, vitamin D-enhancing sun exposure when possible.

### 1.3. Dietary Sources of Vitamin D

A limited number of foods naturally contain vitamin D, especially in quantities that can sustain the Reference Nutrient Intake (RNI) (Table 1) for healthy adults, which is 10 μg/day (400 IU/day) in UK [2]. Each vitamin D metabolite exists in two major forms: D_3_ and D_2_. Vitamin D_3_ can be obtained through consumption of animal products and vitamin D_2_ from plant sources. The foods that contain vitamin D_3_ include fish (e.g., eel, herring, salmon, tuna, cod, sardines, and mackerel), egg yolk, red meat, cheese, dairy products and butter. Vitamin D_2_ can be found in some wild mushrooms, or in white mushrooms if they have been exposed to UV radiation [21]. Some foods, such as milk, cereals, margarine and orange juice, can also be fortified to increase their vitamin D content [22,23,24].

Here, we have performed a questionnaire-based study directly comparing aspects of lifestyle in young and older adults based in Greater Manchester. The aim was to compare dietary and sun exposure behaviours of young and older adults and assess the impact on their vitamin D status.

## 2. Materials and Methods

### 2.1. Volunteers

This pilot study was performed in healthy volunteers, in the Photobiology Unit, Dermatology Research Centre, University of Manchester, based at Salford Royal NHS Foundation Trust, UK. Inclusion criteria were for volunteers to be healthy, ambulant male or female adults of phototype I–III (white Caucasian), aged 18–40 years old and 65–89 years old. These two groups, from Greater Manchester, were recruited through advertisements and the Photobiology Unit database (November 2018–January 2019). They completed the questionnaires/blood sampling in the period of January–March 2019.

Exclusion criteria took account of further studies performed subsequently in these volunteers, and were: history of skin cancer/photosensitivity, use of sunbed/sunbathing within 3 months, taking photoactive medication/bone active therapies, taking vitamin D doses >200 IU (5 μg)/day, taking anticoagulants (including Aspirin, Clopidogrel, Warfarin and Propranolol). The North West Greater Manchester West Research Ethics Committee provided ethical approval (reference 18/NW/0493). This study adhered to the Declaration of Helsinki; all volunteers gave written, informed consent. We had to exclude four potential volunteers. Three of the potential younger volunteers did not fit the skin type inclusion criteria (skin type I–III), while one of the potential older volunteers was undergoing immune-system suppressant with methotrexate, and had to be excluded on the grounds of clinical safety measures.

### 2.2. Skin Type Assessment

Volunteers were assessed for sun-reactive skin type at the Photobiology Unit using a standardised series of questions relating to history of skin responses to sunlight exposure. We used a modified Fitzpatrick phototype assessment [25], as previously described [26]. They are all white Caucasian with a history of sunburn on sunlight exposure, with skin type I being those who sunburn very easily and do not tan, skin type II being those who sunburn easily and tan slightly, and skin type III being those who sunburn sometimes and tan moderately.

### 2.3. Lifestyle Questionnaires

Volunteers completed a four-page questionnaire, adapted from previous Photobiology Unit studies [27,28] to assess their sun exposure and related behaviours. The questionnaire (see Supplementary Materials) was composed of the following sections: (1) work/outdoor activities: employment status, outdoor activities, length of periods outdoors, outdoor hobbies; (2) holidays: number and length of summer and winter holidays, destination (defined as (a) UK destination, (b) outside UK, but same sun exposure as UK, (c) outside UK, but higher sun exposure, (d) holidays in UK and abroad: mixture of a–c), clothing normally worn during the day and time outdoors; (3) sunscreen use: frequency of use of sunscreen and area covered during summer at home (in the UK), on summer and winter holiday; (4) sunbeds: if the volunteer had ever used a tanning sunbed/solarium/UV lamp and if they would ever consider using one in the future. The answers were given a numerical score; the midpoint of the time range was used to calculate mean hours spent outdoors (see Table 2).

### 2.4. Vitamin D Diet Diaries

Volunteers completed an 11-page diet diary in order to assess their dietary intake of vitamin D. The diary involved answering six general questions on diet preferences including following a vegan/vegetarian diet, type of spread and milk used. The remaining pages included a table for a daily record of vitamin D-rich foods intake; the sections were: fortified foods, red meat, cheese, milk, other dairy products, eggs, fat spread and fatty fish. Dietary vitamin D content was assessed according to the sixth edition and integrated data set of McCance and Widdowson’s The Composition of Food [29] and from food-package labelling.

### 2.5. Circulating Vitamin D Analysis

Each participant provided a blood sample for 25OHD analysis. Peripheral blood (approx. 20 mL) was collected in the months January–March 2019, thus vitamin D status was measured close to the winter nadir in the seasonal cycle. On collection, it was separated by centrifugation, and serum samples were stored at −80 °C prior to analysis. Samples were analysed locally at Salford Royal NHS Foundation Trust for routine biochemistry, including renal function and parathyroid hormone (PTH). Serum analysis of circulating total 25(OH)D was performed by LC–MS/MS at the University of East Anglia (UEA). Anonymised samples were transported to the UEA Bioanalytical Facility (BAF) for analysis. The facility is accredited by the Central Pathology Accreditation (CPA) since 2013 (CPA reference number 0868b) and recently accredited for Good Clinical Laboratory Practice (GCLP) (accreditation number 05617) in late 2017. The facility holds the Vitamin D External Quality Assessment Scheme (DEQAS) certificates for 25(OH)D measurements.

### 2.6. Statistical Software

The results of lifestyle questionnaires and vitamin D diet diaries were analysed using GraphPad Prism statistical software (version 8.4.3, 10 June 2020). 25(OH)D results were assessed for normality using Shapiro–Wilk test and then analysed using an unpaired t-test; diet diary data were analysed using two-way ANOVA (GraphPad Prism statistical software, as above). The combination of categorical data and small sample size did not allow for statistical comparison using chi-squared test (or Fisher’s exact test) of the lifestyle questionnaire data. This set of data was analysed using descriptive statistics. The collection of lifestyle questionnaires/diet diaries and blood sampling were performed alongside skin biopsy collection for 7-dehydrocholesterol content analysis, with the latter determining the overall sample size of this study.

## 3. Results

### 3.1. Volunteers

A total of 25 volunteers were recruited—14 in the younger age group and 11 in the older age group. One participant from the 18–40 year cohort withdrew his participation. All available data were used in this analysis. Table 3 contains data on volunteers’ gender, age, skin type, BMI, total 25(OH)D concentration and employment. The concentration of 25(OH)D was lower in the younger age group than in the older age group, contrary to traditional expectations [8,30]. However, there was no statistical difference when groups were compared (*p* = 0.1592; t = 1.457, df = 22).

### 3.2. Occupation

A total of 85% (*n* = 11) of the young volunteers (18–40 years old) and 18% (*n* = 2) older (65–89 years old) were employed. Of those who were working, two (100%) in the older group and one (9%) in the younger group worked in administrative and secretarial occupations. In the younger group, one (9%) worked in managerial, four (36%) in associate professional and technical occupations, one (9%) in skilled trades, two (18%) in sales and customer service, one (9%) in process, plant and machine operatives and one (9%) was a student with part-time job(s). All employment was considered predominantly indoor occupation.

### 3.3. Time Spent Outdoors in the UK

**Figure 1:**
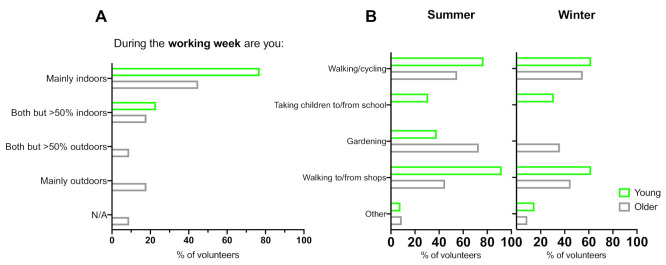
(**A**) Percentage responses of younger and older volunteers who consider themselves to spend time during the working week: mainly indoors, mainly outdoors, or both, but >50% indoors or >50% outdoors; (**B**) percentage responses of younger and older volunteers’ activities during the working week when outdoors for short regular periods in summer and winter (multiple choice question).

Figure 1 shows outdoor activities during the working week. All subjects in both groups reported spending regular short periods outdoors during the working week, with walking/cycling (younger: 77%; older: 55%) and walking to/from shops (younger: 92%; older: 45%) being the most common outdoor activities. A total of 77% of the younger group reported being ‘mainly indoors’ compared to 45% of the older group, the remaining 23% of younger and 18% of older group said they were ‘both indoors and outdoors, but >50% indoors’, whilst 18% of the older group reported being ‘mainly outdoors’ and 9% ‘both indoors and outdoors, but >50% outdoors’. Additionally, 23% of younger and 55% of older volunteers said they had regular outdoor hobbies/activities.

**Figure 2:**
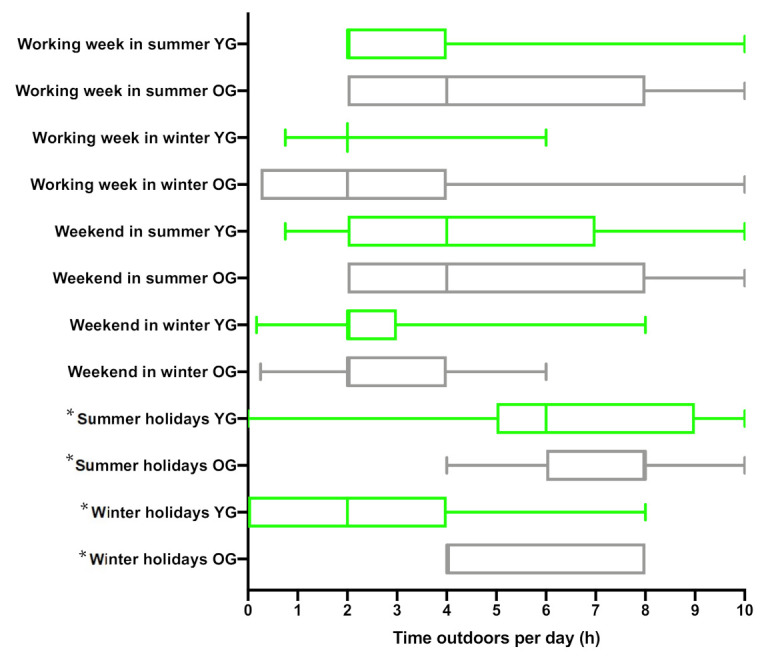
Time spent outdoors in younger (YG) and older (OG) groups of volunteers during the working week in summer and winter at home in the UK, weekend in summer at home in the UK, summer and winter holidays. Plot shows range (whiskers), median (thick lines) and lower and upper quartiles (boxes) of number of hours spent outdoors per day. * The length of the holiday was given a value of zero (0) if volunteers reported not going on holidays.

In summer during the working week, younger subjects spent shorter periods outdoors per day compared to older subjects (median 2 h, range 2–4; median 4 h, range 2–8, respectively), while there was no difference during weekends in summer (younger: median 4 h, range 2–7, older: median: 4 h, range 2–8), see Figure 2. Both groups spent a similar amount of time outdoors during the working week in winter (younger: median 2 h, range 2–2, older: median 2 h, range 0.25–4) and weekend in winter (younger: median 2 h, range 2–3, older: median: 2 h, range 2–4). It is important to note that winter days are colder and shorter, and do not provide enough UVB to produce biologically relevant quantities of vitamin D.

### 3.4. Holiday Behaviour

**Figure 3:**
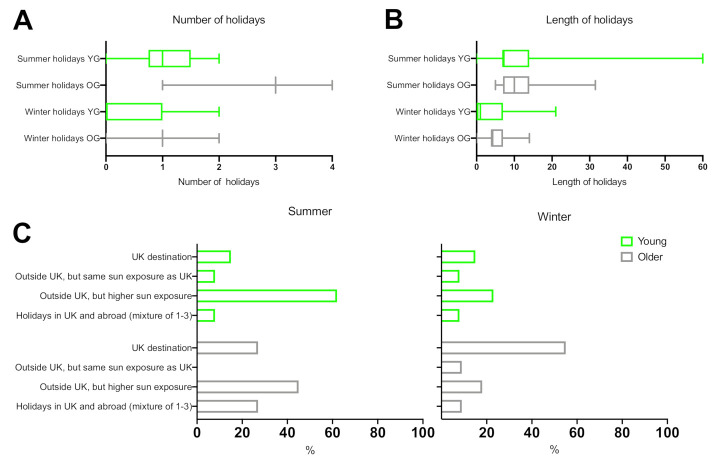
(**A**) Number of winter and summer holidays taken by younger (YG) and older groups (OG). (**B**) Length of winter and summer holidays in younger (YG) and older groups (OG). Plots A and B show range (whiskers), median (thick lines) and lower and upper quartiles (boxes) of number and length of winter and summer holidays. (**C**) Percentages of younger and older volunteers responses showing the type of holiday destination they take during summer and winter holidays.

Overall, 85% of young and 100% of older volunteers said they go on holiday each year (Figure 3). Older subjects took a greater number of both summer and winter holidays each year (median 3, range 3–3; median 1, range 1–1, respectively) than young subjects (median 1, range 0.75–1.5; median 0, range 0–1, respectively). The length of summer holiday was similar in both groups (older: median 10 days, range 7–14 days; young: median 7 days, range 7–14 days), while length of winter holiday was higher in the older group than young (older: median 4 days, range 4–7 days; younger: median 1 day, range 0–4 days). Both groups spend similar amounts of time outdoors on summer holiday (older: median 8 h, range 6–8 h; young: median 6 h, range 5–9 h), but the older group reported to spend more time outdoors on winter holiday (older: median 4 h, range 4–8 h; young: median 2 h, range 0–4 h; Figure 2). A total of 62% of young and 45% of older volunteers reported going on summer holiday outside the UK, where sun exposure is higher, while more older volunteers reported spending summer holidays in the UK (27%) compared to the young (15%) group.

### 3.5. Holiday Clothing Choices

**Figure 4:**
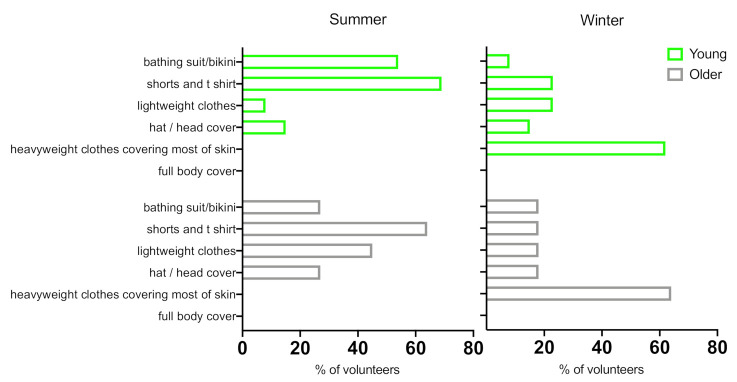
Normal clothing worn during the day whilst on summer and winter holidays by younger and older volunteers.

Figure 4 shows typical clothing choices whilst on holiday. When on summer holiday more young volunteers reported wearing ‘bathing suit/bikini’ (54%) than older (27%), while the majority of subjects in both groups normally wore ‘shorts and t-shirt’ during the day (69% and 64%, respectively). More volunteers in the older group wore ‘hat/head cover’ than in young group (27% vs. 15%) during summer holiday. During the winter holiday, only 8% of younger and 18% of older adults normally wore ‘bathing suit/bikini’ and the majority of volunteers in both groups wore ‘heavyweight clothes covering most of skin’ (young: 62%; older: 64%). ‘Hat/head cover’ was worn by 15% of young and 18% of older volunteers.

### 3.6. Sunscreen Use

**Figure 5:**
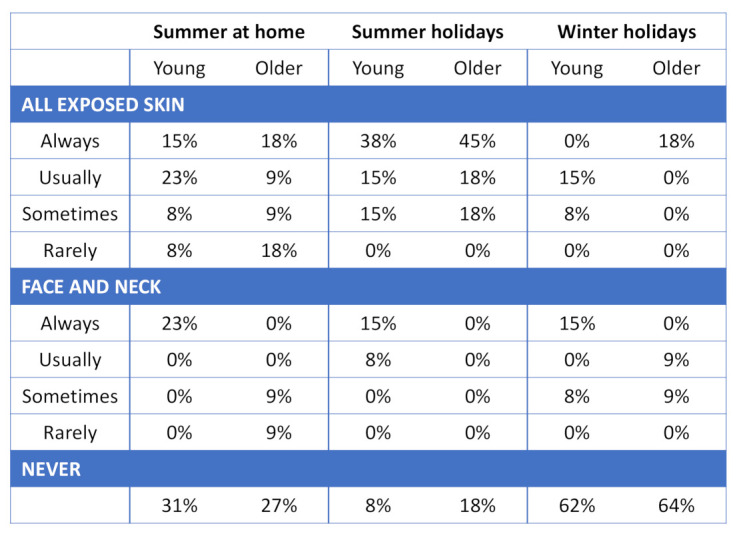
Responses to sunscreen use multiple choice question expressed as percentages.

Sunscreen use was greater during summer holidays than during winter holidays in both groups (Figure 5) with 38% of young and 45% older volunteers always wearing sunscreen on all exposed skin during summer holidays compared to 0% young and 18% older volunteers during winter holidays. A total of 15% of young and 18% of older volunteers reported always wearing sunscreen on all exposed skin during summer at home in the UK. A total of 31% of young and 27% of older volunteers reported never wearing a sunscreen in summer at home in the UK. Additionally, 8% of young and 18% of older volunteers never wore sunscreen during summer holidays and 62% of young and 64% of older volunteers used no sunscreen during winter holidays.

### 3.7. Sunbed Use

A total of 46% (*n* = 6; 4 male, 2 female) of young and 27% (*n* = 3; 1 male, 2 female) of older subjects stated that they had ‘ever used a sunbed’, with 3.6 being the mean number of times used in the younger group and 1.0 in the older group. Only two volunteers (15%) in the younger and one volunteer (9%) in the older group reported that they would use a sunbed again in the future.

### 3.8. Dietary Vitamin D Intake

**Figure 6:**
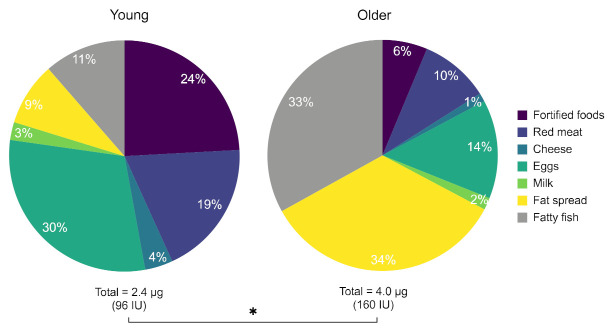
Average dietary vitamin D intake in μg (and IU) per day in young and older groups of volunteers. * indicates *p* value of < 0.05 (significant).

Average daily dietary intake of vitamin D was significantly different between the younger (2.4 μg) and older group (4.0 μg) (*p* = 0.0189; F (6, 154) = 2624), Figure 6. There was also a statistically significant difference in multiple comparison tests between groups in the intake of fat spread (*p* = 0.017) and fatty fish (*p* = 0.040). However, in both groups daily intake was considerably lower than the current vitamin D RNI for UK adults of 10 μg/day (400 IU/day), which is the amount estimated to achieve a serum 25(OH)D concentration ≥25 nmol/L during winter in 97.5% of the population [2]. Interestingly, 67% of overall vitamin D intake in the older group was from fatty fish (33%) and fat spread (34%), whilst the two groups which provided most of the vitamin D intake (54%) in young volunteers were fortified foods (24%) and eggs (30%).

### 3.9. Vitamin D Supplements

During screening two volunteers in the young and four in the older group reported usually taking supplements containing vitamin D. One of the older volunteers continued taking their low dose supplement during this study, as permitted by the study exclusion criteria; this was within the prescription medicine Adcal D_3_ (200 IU vitamin D daily).

## 4. Discussion

Traditional beliefs regarding the vitamin D status of older adults [8,10,30] and their lifestyle behaviours in general might no longer be correct [15,16,17]. The discrepancy between our results and the traditional view that older adults have low vitamin D status can be explained by differences in the populations being tested; in the past, institutionalised and frail older adults were often recruited. Moreover, life expectancy has increased by 7.5 years since 1950. Much of this is estimated to be due to improved medical care [31], while improved physical activity also has a well-established correlation with health and life expectancy [32,33,34], and campaigns promoting physical activity have been shown to have effect on increases in activity levels [35,36] in the population. With behavioural shifts occurring, there is a need to re-consider if the traditional definition of ‘old age’ needs updating [37,38,39].

While the vast majority of younger volunteers reported that they are ‘mainly indoors’ during the working week, less than half (45%) of older volunteers selected that answer. Moreover, no younger volunteers, but 18% of older volunteers, said they are ‘mainly outdoors’. This information was also reflected in their estimates of how much time (in hours) they spend outdoors during the working week in summer—younger subjects range was 2 to 4 h; while older subjects range was 2 to 8 h. These self-reported behaviours might be indicating that older adults have higher casual sun exposure during the working week, facilitated by the fact that many of them are no longer employed. The groups’ holiday behaviour was also different. A total of 100% of older and 85% of young volunteers reported going on holiday each year. Furthermore, older subjects took more of both summer (median 3) and winter (median 1) holidays than younger subjects (median 1, median 0, respectively). Interestingly, older volunteers spend more time outside on winter holiday than the younger group (range 4–8 h, range 0–4 h, respectively) and when travelling to a destination with a higher sun exposure than the UK, it would influence their vitamin D status. This is also echoed in their clothing choices during the winter holiday. A total of 18% of older compared to 8% of young subjects reported wearing ‘bathing suit/bikini’ during their winter holiday, implying they were somewhere warm at lower latitudes.

Overall, our data show that healthy 65+ year olds spend more time outdoors in summer than working-age adults and have more outdoor hobbies. They also go on holiday more often, which when travelling to high sun exposure destinations would boost their vitamin D status.

While both groups’ oral intake of vitamin D was below the RNI of 10 ug/day [2], the dietary vitamin D intake of the older adults (4.0 μg) we studied was significantly higher than that of the younger group (2.4 μg). There was a contrast between the food groups supplying most of the dietary vitamin D intake in younger and older volunteers. The majority of overall vitamin D intake in the older group came from fatty fish (33%) and fat spread (33%). In the younger group, the top two categories were fortified foods (24%) and eggs (30%). Fatty fish is known for having the highest concentration of vitamin D in foodstuff (see Table 1)—but only 11% of dietary vitamin D intake came from fatty fish in young subjects.

Moreover, while oral vitamin D supplements of above 200 IU/d were an exclusion criterion in this study, we also know from the initial screening that more of the older adults (*n* = 4) than younger adults (*n* = 2) took vitamin D-containing supplements. Hence, there was evidence that the vitamin D intake was higher both from the sun exposure and oral routes in the older group.

Our findings with regard to vitamin D status are consistent with this evidence. In our study, winter 25(OH)D concentrations were not significantly different between older (56.9 ± 24.9 nmol/L) and younger groups (43.2 ± 21.1 nmol/L). The sample size may be a limiting factor here, potentially missing a significant finding; however, we can decisively state that the vitamin D status of the older group was *not* lower than that of the younger group, and matched our questionnaire and diary findings. However, as this study was not designed with statistical power in mind this is one of its limitations: a difference in 25(OH)D twice that observed would have been required for significance in this small sample size.

Although we saw a number of lifestyle differences between age groups, their sun protective measures were similar. Despite efforts of government organisations, health boards and charities to inform the public about safe enjoyment of the sun [40,41], 31% of young and 27% of older volunteers still reported that they never wear a sunscreen in summer at home in the UK, and 8% and 18% did not during summer holiday, respectively. Additionally, only 15% of young and 27% of older volunteers wore hat/head cover during summer holiday. While a ‘little and often’ approach to sun exposure is very important for vitamin D production, the balance between safe and dangerous sun exposure is poorly understood and not adhered to by public [7]. These results indicate the further need to emphasise and educate the public in the risk/benefit balance of sun exposure. Our data also suggest that educating older adults about physical activity and diet may be beneficial in order for them to maintain good vitamin D status.

## 5. Conclusions

In this preliminary study, we compared sun exposure behaviours and dietary vitamin D intake of a sample of young and older adults in the UK, and investigated how this may be impacting their vitamin D status. Our data suggest that healthy older adults’ vitamin D status was at least as good as that of younger adults, while their sun protective behaviours were similarly poor. These interesting pilot findings indicate the need for larger studies, powered to enable generalisation to the UK population, to further evaluate our understanding of vitamin D status in today’s older adults. Furthermore, the authors suggest the need for further study investigating the influence of outdoor activity and a vitamin D-rich diet with increasing age across the broader post-retirement community.

## Figures and Tables

**Table 1:** Selected foods and their vitamin D content per 100 g [21,23].

Food	Vitamin D (μg/100 g)
Eel	25.6
Herring	15.4
Salmon	12.4
Egg yolk (egg)	7.8 (2.8)
Tuna	7.2
Cod	7.0
Milk, fortified	1.3–2.0
Beef liver	0.8
Cheddar cheese	0.3–0.6
Butter	0.3–1.5
Milk, whole, unfortified	0.1

**Table 2:** Scoring system for lifestyle questionnaire responses of time-outdoors.

Hours Outdoors—Available Answers	Score
30 min or less	1
>30 min to <1 h	2
1 h to <3 h	3
3 h to <5 h	4
5 h to <7 h	5
7 h to <9 h	6
9 h or more	7

**Table 3:** Patient Demographics. Mean values unless otherwise stated. Healthy BMI range 18.5–25 kg/m^2^. Deficiency of vitamin D defined as below 25 nmol/L (10 ng/mL); sufficiency defined as 50 nmol/L (30 ng/mL) and above, of 25(OH)D [2]. Skin type I, II, III based on Fitzpatrick phototype assessment [25].

Group	Young (18–40 Years Old)	Older (65–89 Years Old)
Participants, *n*	13	11
Gender: male, *n* (%)	7 (54)	6 (55)
Gender: female, *n* (%)	6 (46)	5 (45)
Mean 25(OH)D [nmol/L] (±SD)	43.2 (21.1)	56.9 (24.9)
Mean BMI [kg/m^2^] (±SD)	27.1 (4.6)	27.1 (5.4)
Skin type I, II, III	3, 3, 7	2, 2, 7
Mean age (±SD)	30.1 (6.2)	70.8 (4.6)
Employed, *n* (%)	11 (85)	2 (18)

## Data Availability

Data available on request from the corresponding author.

## Supplementary Materials

The following are available online at https://www.mdpi.com/1660-4601/18/7/3364/s1, Document S1: Diet Diary, Document S2: Lifestyle Questionnaire.
