# Supplementary material for: Older Adults Who Spend More Time Outdoors in Summer and Have Higher Dietary Vitamin D Than Younger Adults Can Present at Least as High Vitamin D Status: A Pilot Study
Source: Int J Environ Res Public Health. 2021 Mar 24;18(7):3364. doi: 10.3390/ijerph18073364 (PMC8037349; doi:10.3390/ijerph18073364)
Supplement: Supplementary file 1 [file ijerph-18-03364-s001.zip › Lifestyle questionnaire v1_18th March 2018.docx]

**
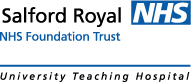
**
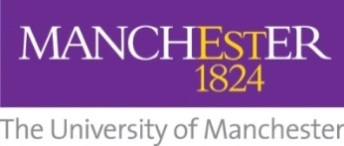


**Initials** **Participant no**

**LIFESTYLE QUESTIONNAIRE**

**Age-related ability to synthesise vitamin D in the skin on exposure to sunlight**

**Work/Outdoor Activities**

In the first set of questions we are considering the working day (8am-6pm). If you do not have a full time job then simply select the answer that best matches where you are at particular times of day or week. Do not include holiday periods. Summer is defined as April – Sept, winter as Oct – March.

1. **Are you employed?** Yes  No 

*If yes:*

(a) What is your occupation? .................................................

(b) Is it full-time or part-time? FT PT (Please circle)

**Are you doing regular voluntary work?** Yes  No 

If yes:

(a) What is it that you do? .................................................

(b) Is it full-time or part-time? FT PT (Please circle)

2. **During the working week are you**: (Please tick *one* box only):

**tick**

| Mainly indoors |  |
| --- | --- |
| Mainly outdoors |  |
| Both but more than 50% **i*ndoors*** |  |
| Both but more than 50% ***outdoors*** |  |

3. **Do you spend regular short periods outdoors?** (Please tick *all* that apply)

.

| **In Summer** | **Tick** |  |  | **In Winter** | **Tick** |
| --- | --- | --- | --- | --- | --- |
| Walking / cycling |  |  |  | Walking / cycling |  |
| Taking children to/from school |  |  |  | Taking children to/from school |  |
| Gardening |  |  |  | Gardening |  |
| Walking to / from shops |  |  |  | Walking to / from shops |  |
| Other (please specify below) |  |  |  | Other (please specify below) |  |

4. **Please estimate how many hours you spend outdoors per day during the working week on average.**

(Please tick *one* box only)

| **In Summer** | **Tick** |  | **In Winter:** | **Tick** |
| --- | --- | --- | --- | --- |
| ½ hr or less |  |  | ½ hr or less |  |
| More than ½ hr |  |  | More than ½ hr |  |
| From 1 hr to less than 3 hrs |  |  | From 1 hr to less than 3 hrs |  |
| From 3 hrs to less than 5 hrs |  |  | From 3 hrs to less than 5 hrs |  |
| From 5 hrs to less than 7 hrs |  |  | From 5 hrs to less than 7 hrs |  |
| From 7 hrs to less than 9 hrs |  |  | From 7 hrs to less than 9 hrs |  |
| 9 hrs or more |  |  | 9 hrs or more |  |

**If you answered “½ hr or less” can you please estimate how many minutes you spend outdoors per day during the working week?**

………………………………………………………………………………………………………………………………………………………………………………………………………..

5. **Do you have regular outdoor hobbies/activities?** Yes  No 

If **yes**, could you please name these: ……………………………………………………………………………………………………………………………………………………………………………………………………………………

6. **Please estimate how many hours you spend outdoors per day at the weekend**

(Please tick *one* box only)

| **In Summer** | **Tick** |  | **In Winter:** | **Tick** |
| --- | --- | --- | --- | --- |
| ½ hr or less |  |  | ½ hr or less |  |
| More than ½ hr |  |  | More than ½ hr |  |
| From 1 hr to less than 3 hrs |  |  | From 1 hr to less than 3 hrs |  |
| From 3 hrs to less than 5 hrs |  |  | From 3 hrs to less than 5 hrs |  |
| From 5 hrs to less than 7 hrs |  |  | From 5 hrs to less than 7 hrs |  |
| From 7 hrs to less than 9 hrs |  |  | From 7 hrs to less than 9 hrs |  |
| 9 hrs or more |  |  | 9 hrs or more |  |

**If you answered “½ hr or less” can you please estimate how many minutes you spend outdoors per day at the weekend? ………………………………………………**

**…………………………………………………………………………………………………..**

**…………………………………………………………………………………………………..**

**HOLIDAYS** *Please note- We define a holiday as 3 or more full days spent away from your home location i.e. one day travelling, 3 + days away and one day travelling back. Please do not include shorter breaks.*

**7. Do you go away on holiday each year? Yes  No **

**8. How many times a year do you take a holiday in the summer months**

**(April to September)?** ......................................................................................................

**9. Where do you typically go in summer (or where did you go last summer)?**

……………………………………………………………………………………………………

**10. How long is your usual summer holiday?** ……………………………………………

**11. How many times a year do you take a holiday in the winter months**

**(October to March)?** ……………………………………………………………………………

**12. Where do you typically go in winter (or where did you go last winter)?**

……………………………………………………………………………………………………

**13. How long is your usual winter holiday?** ………………………………………………

**14. When you are on holiday how do you normally dress during the day?**

| **In Summer** | **Tick** |  | **In Winter:** | **Tick** |
| --- | --- | --- | --- | --- |
| Bathing suit / bikini |  |  | Bathing suit / bikini |  |
| Shorts and T-shirt (or equivalent) |  |  | Shorts and T-shirt (or equivalent) |  |
| Lightweight clothes covering more skin than shorts and T shirt |  |  | Lightweight clothes covering more skin than shorts and T shirt |  |
| Head covered /Hat |  |  | Head covered /Hat |  |
| Heavyweight clothes covering most of skin |  |  | Heavyweight clothes covering most of skin |  |
| Full body cover (e.g.: for skiing) |  |  | Full body cover (e.g.: for skiing) |  |

15. **How many hours a day do you spend outdoors when on your holiday?**

| **In Summer** | **Tick** |  | **In Winter:** | **Tick** |
| --- | --- | --- | --- | --- |
| ½ hr or less |  |  | ½ hr or less |  |
| More than ½ hr |  |  | More than ½ hr |  |
| From 1 hr to less than 3 hrs |  |  | From 1 hr to less than 3 hrs |  |
| From 3 hrs to less than 5 hrs |  |  | From 3 hrs to less than 5 hrs |  |
| From 5 hrs to less than 7 hrs |  |  | From 5 hrs to less than 7 hrs |  |
| From 7 hrs to less than 9 hrs |  |  | From 7 hrs to less than 9 hrs |  |
| 9 hrs or more |  |  | 9 hrs or more |  |

**SUNSCREEN USE**

16. **Do you wear sunscreen?** (Please tick for a, b, and c if applicable)

**a) In summer at home:**

**tick**

| Yes, always, all exposed skin |  |
| --- | --- |
| Yes, always, face and neck |  |
| Usually, all exposed skin |  |
| Usually, face and neck |  |
| Sometimes, all exposed skin |  |
| Sometimes, face and neck |  |
| Rarely, all exposed skin |  |
| Rarely, face and neck |  |
| Never |  |

**b) On a summer holiday:**

**tick**

| Yes, always, all exposed skin |  |
| --- | --- |
| Yes, always, face and neck |  |
| Usually, all exposed skin |  |
| Usually, face and neck |  |
| Sometimes, all exposed skin |  |
| Sometimes, face and neck |  |
| Rarely, all exposed skin |  |
| Rarely, face and neck |  |
| Never |  |

**c) On a winter holiday:**

**tick**

| Yes, always, all exposed skin |  |
| --- | --- |
| Yes, always, face and neck |  |
| Usually, all exposed skin |  |
| Usually, face and neck |  |
| Sometimes, all exposed skin |  |
| Sometimes, face and neck |  |
| Rarely, all exposed skin |  |
| Rarely, face and neck |  |
| Never |  |

**SUNBEDS**

**Have you ever used a sunbed / solarium / UV lamp?**

If **yes**, how often? ………………………………………….

**Would you use a sunbed / solarium / UV lamp in the future?** Yes  No 
