# Supplementary material for: Older Adults Who Spend More Time Outdoors in Summer and Have Higher Dietary Vitamin D Than Younger Adults Can Present at Least as High Vitamin D Status: A Pilot Study
Source: Int J Environ Res Public Health. 2021 Mar 24;18(7):3364. doi: 10.3390/ijerph18073364 (PMC8037349; doi:10.3390/ijerph18073364)
Supplement: Supplementary file 1 [file ijerph-18-03364-s001.zip › Vitamin D Diet Diary_v1 8th March 2018.doc]

University of Manchester

**Age-related ability to synthesise vitamin D in the skin on exposure to sunlight**

Diet Diary

Month:

W/c: Monday dd/mmm/yyyy

**University of Manchester REC reference: ________**

**IRAS Reference:** 235670

**Photobiology Unit**

Initials:Participant Number

We would like to know how much vitamin D you are getting from your diet. Please tick where appropriate.

**1a.**. Do you ever take supplements that contain vitamin D?

(E.g. multivitamin, fish oils)

Yes	🞏	No	🞏

**1b.**. Do you have vitamin D prescribed by your doctor? (E.g Adcal D_3_, HUX D_3_ , Alendronic acid. Colecalciferol)

Yes	🞏	No	🞏

**2**. **If either answer above is yes,** please give us details of the medicine / supplement, and how much vitamin D it contains (if you don’t know please bring the packaging along for us to look at.)

…………………………………………………………………………………………………

…………………………………………………………………………………………………

**3**. Are you a vegetarian?

Yes	🞏	No	🞏

**4**. Are you a vegan?

Yes	🞏	No	🞏

**5**. What spread do you usually use on bread / vegetables?

None	🞏

Butter (E.g. Country life)	🞏………………………….

Other spread	🞏………………………….

(E.g. Utterly Butterly / Flora Light)

Are they Vitamin D fortified?	Yes	🞏	No	🞏

Please give details	…………………………………………………………

**6**. What type of milk do you drink or use?

Whole milk (full fat)	🞏

Semi-skimmed	🞏

Skimmed	🞏

Vitamin D fortified	🞏

(Please give brand)	……………………………………………………………

Here is an example of how to fill in your diet diary:

- Within the table overleaf fill in as much information as possible. Please fill in the amount of certain foods you have eaten each day. You can fill it in by meal (sometimes easiest to remember) or simply as a total for the whole day.
- Please estimate portions, use common measures to help you (e.g. teaspoon (tsp), tablespoon (tbsp), cup, small bowl, large bowl, you can estimate how much of a full pack or tin you ate e.g. half can tinned tuna or 92.5g use other dimensions e.g. steak = 2” x 4” x 1” (or 5cm x 10 cm x 2.5 cm). You can use any units that you are familiar with (lb, oz, g, kg, pint, litre, ml, inches and cm).
- We ONLY need to know when you ate food containing vitamin D, or vitamin D fortified (added) NOT everything you ate during the day.

Explanation of example over leaf, please use a page per day.

During the day our volunteer ate:

- Breakfast;

A bowl of cereal with milk.

- Lunch;

A tuna sandwich with thick filling and butter on brown bread.

- Dinner;

Steak with new potatoes, butter and peas.

Milk was used in tea and coffee throughout the day.

We have given the above as an example; we do not expect you to fill in every box every day. We want you to eat your normal diet, without changing anything to “look good” in the diary. The results will be anonymous.

*Please remember to bring this to your appointment next week*

Many thanks for your time.

| **Day:** Monday | **** Example Page**** |
| --- | --- |
| **Date:** |  |

| **Food** | **Product & brand** | **Quantity** | **Not eaten today^*^** |
| --- | --- | --- | --- |
| Fortified foods  (e.g. cereal, certain mushrooms, certain milk) | Kellogs fruity flakes cereal | 30g |  |
| Vitamin D Supplements (e.g. multivitamin, cod liver oil) |  |  |  |
| Red Meat  (e.g. Beef, lamb, pork, liver) | Steak | 2” x 4” x 1” |  |
| Cheese e.g. Swiss cheese.  Any dairy products such as yoghurt and ice cream |  |  | **X** |
| Eggs  (e.g. whole boiled, fried  Omelette or quiche) |  |  | **X** |
| Total milk used in the day (inc drinks, sauces) | Semi skimmed milk - Aldi | ¾ pint |  |
| Total fat spread used for the day | Butter -Lidl | 2 gm |  |
| Fatty fish  e.g. Sardines, mackerel, tuna, Herring. | Tinned Tuna in Brine Tesco  own brand | Half a tin or 92.5gms |  |
|  | * Please put a **X** at the end of the row for foods not eaten today | | |

**Day 1:**

**Date:**

| **Food** | **Product & brand** | **Quantity** | **Not eaten today^*^** |
| --- | --- | --- | --- |
| Fortified foods  (e.g. cereal) |  |  |  |
| Vitamin D Supplements (e.g. multivitamin, fish oil) |  |  |  |
| Red Meat  (e.g. Beef, lamb, pork, liver bacon) |  |  |  |
| Cheese e.g. Swiss cheese. |  |  |  |
| Dairy products such as yoghurt and ice cream |  |  |  |
| Eggs: Small/medium/large (whole boiled, fried  Omelette, quiche, other) |  |  |  |
| Total milk used in the day (inc drinks, sauces) |  |  |  |
| Total fat spread used for the day |  |  |  |
| Fatty fish  e.g. Sardines, mackerel, tuna, Herring. |  |  |  |
|  | * Please put a **X** at the end of the row for foods not eaten today | | |

**Day 2;**

**Date:**

| **Food** | **Product & Brand** | **Quantity** | **Not eaten today^*^** |
| --- | --- | --- | --- |
| Fortified foods  (e.g. cereal) |  |  |  |
| Vitamin D Supplements (e.g. multivitamin, fish oil) |  |  |  |
| Red Meat  (e.g. Beef, lamb, pork, liver bacon) |  |  |  |
| Cheese e.g. Swiss cheese. |  |  |  |
| Dairy products such as yoghurt and ice cream |  |  |  |
| Eggs: Small/medium/large (whole boiled, fried  Omelette, quiche, other) |  |  |  |
| Total milk used in the day (inc drinks, sauces) |  |  |  |
| Total fat spread used for the day |  |  |  |
| Fatty fish  e.g. Sardines, mackerel, tuna, Herring. |  |  |  |
|  | * Please put a **X** at the end of the row for foods not eaten today | | |

**Day 3:**

**Date:**

| **Food** | **Product & Brand** | **Quantity** | **Not eaten today^*^** |
| --- | --- | --- | --- |
| Fortified foods  (e.g. cereal) |  |  |  |
| Vitamin D Supplements (e.g. multivitamin, fish oil) |  |  |  |
| Red Meat  (e.g. Beef, lamb, pork, liver bacon) |  |  |  |
| Cheese e.g. Swiss cheese. |  |  |  |
| Dairy products such as yoghurt and ice cream |  |  |  |
| Eggs: Small/medium/large (whole boiled, fried  Omelette, quiche, other) |  |  |  |
| Total milk used in the day (inc drinks, sauces) |  |  |  |
| Total fat spread used for the day |  |  |  |
| Fatty fish  e.g. Sardines, mackerel, tuna, Herring. |  |  |  |
|  | * Please put a **X** at the end of the row for foods not eaten today | | |

**Day 4:**

**Date:**

| **Food** | **Product & Brand** | **Quantity** | **Not eaten today^*^** |
| --- | --- | --- | --- |
| Fortified foods  (e.g. cereal) |  |  |  |
| Vitamin D Supplements (e.g. multivitamin, fish oil) |  |  |  |
| Red Meat  (e.g. Beef, lamb, pork, liver bacon) |  |  |  |
| Cheese e.g. Swiss cheese. |  |  |  |
| Dairy products such as yoghurt and ice cream |  |  |  |
| Eggs: Small/medium/large (whole boiled, fried  Omelette, quiche, other) |  |  |  |
| Total milk used in the day (inc drinks, sauces) |  |  |  |
| Total fat spread used for the day |  |  |  |
| Fatty fish  e.g. Sardines, mackerel, tuna, Herring. |  |  |  |
|  | * Please put a **X** at the end of the row for foods not eaten today | | |

**Day 5:**

**Date:**

| **Food** | **Product & Brand** | **Quantity** | **Not eaten today^*^** |
| --- | --- | --- | --- |
| Fortified foods  (e.g. cereal) |  |  |  |
| Vitamin D Supplements (e.g. multivitamin, fish oil) |  |  |  |
| Red Meat  (e.g. Beef, lamb, pork, liver bacon) |  |  |  |
| Cheese e.g. Swiss cheese. |  |  |  |
| Dairy products such as yoghurt and ice cream |  |  |  |
| Eggs: Small/medium/large (whole boiled, fried  Omelette, quiche, other) |  |  |  |
| Total milk used in the day (inc drinks, sauces) |  |  |  |
| Total fat spread used for the day |  |  |  |
| Fatty fish  e.g. Sardines, mackerel, tuna, Herring. |  |  |  |
|  | * Please put a **X** at the end of the row for foods not eaten today | | |

**Day 6:**

**Date:**

| **Food** | **Product & Brand** | **Quantity** | **Not eaten today^*^** |
| --- | --- | --- | --- |
| Fortified foods  (e.g. cereal) |  |  |  |
| Vitamin D Supplements (e.g. multivitamin, fish oil) |  |  |  |
| Red Meat  (e.g. Beef, lamb, pork, liver bacon) |  |  |  |
| Cheese e.g. Swiss cheese. |  |  |  |
| Dairy products such as yoghurt and ice cream |  |  |  |
| Eggs: Small/medium/large (whole boiled, fried  Omelette, quiche, other) |  |  |  |
| Total milk used in the day (inc drinks, sauces) |  |  |  |
| Total fat spread used for the day |  |  |  |
| Fatty fish  e.g. Sardines, mackerel, tuna, Herring. |  |  |  |
|  | * Please put a **X** at the end of the row for foods not eaten today | | |

**Day 7:**

**Date:**

| **Food** | **Product & Brand** | **Quantity** | **Not eaten today^*^** |
| --- | --- | --- | --- |
| Fortified foods  (e.g. cereal) |  |  |  |
| Vitamin D Supplements (e.g. multivitamin, fish oil) |  |  |  |
| Red Meat  (e.g. Beef, lamb, pork, liver bacon) |  |  |  |
| Cheese e.g. Swiss cheese. |  |  |  |
| Dairy products such as yoghurt and ice cream |  |  |  |
| Eggs: Small/medium/large (whole boiled, fried  Omelette, quiche, other) |  |  |  |
| Total milk used in the day (inc drinks, sauces) |  |  |  |
| Total fat spread used for the day |  |  |  |
| Fatty fish  e.g. Sardines, mackerel, tuna, Herring. |  |  |  |
|  | * Please put a **X** at the end of the row for foods not eaten today | | |

Please bring the completed diary back NEXT WEEK

Thank you
